# Recent Advances in Aptamer-Based Applications in Cardiology

**DOI:** 10.3390/ijms27062580

**Published:** 2026-03-11

**Authors:** Aleksandra Kosinova, Tatiana Zamay, Yury Glazyrin, Olga Kolovskaya, Natalia Luzan, Ulyana Beloshedova, Marina Petrova, Yury Grinshtein, Maxim Berezovski, Anna Kichkailo

**Affiliations:** 1 Laboratory for Digital Controlled Drugs and Theranostics, Federal Research Center “Krasnoyarsk Science Center of the Siberian Branch of the Russian Academy of Science”, 660036 Krasnoyarsk, Russia; akosinova@krasgmu.ru (A.K.); uis_70@mail.ru (T.Z.); yury.glazyrin@gmail.com (Y.G.); olga.kolovskaya@gmail.com (O.K.); natalija.luzan@yandex.ru (N.L.); ulya.leggy@mail.ru (U.B.); azamay@krasgmu.ru (A.K.); 2 Therapeutic Department, Prof. V.F. Voino-Yasenetsky Krasnoyarsk State Medical University, 660022 Krasnoyarsk, Russia; grinstein.yi@mail.ru; 3 Laboratory of Biomolecular and Medical Technologies, Prof. V.F. Voino-Yasenetsky Krasnoyarsk State Medical University, 660022 Krasnoyarsk, Russia; 4 Department of Outpatient Therapy and Family Medicine, Prof. V.F. Voino-Yasenetsky Krasnoyarsk State Medical University, 660022 Krasnoyarsk, Russia; stk99@yandex.ru; 5 Department of Chemistry and Biomolecular Sciences, University of Ottawa, Ottawa, ON K1N6N5, Canada

**Keywords:** aptamers, cardiovascular diseases, drug delivery, nanomaterials, thrombosis

## Abstract

Aptamers, short single-stranded DNA or RNA oligonucleotides, are emerging as transformative tools in cardiology for the diagnosis, treatment, and theranostics of cardiovascular diseases (CVDs). This review highlights their dual utility. In diagnostics, aptamers enable the construction of highly sensitive biosensors for key cardiac biomarkers (e.g., troponins, myoglobin, C-reactive protein, natriuretic peptides), outperforming conventional assays and enabling early detection and point-of-care testing. Therapeutically, aptamers offer targeted, controllable, and reversible anticoagulation, as demonstrated by clinical-stage candidates like BT200 (anti-vWF) and NU172 (anti-thrombin), whose action can be rapidly reversed with antidote oligonucleotides. Furthermore, aptamers serve as precision delivery vehicles (e.g., Gint4.T, RNA-Apt30) for transporting therapeutic peptides or nucleic acids specifically to cardiomyocytes. Recent integration with nanomaterials (quantum dots, graphene, liposomes, DNA origami) has led to advanced biosensing and drug delivery platforms. Despite challenges like stability and the polyethylene glycol (PEG) immunogenicity, ongoing clinical trials underscore the significant potential of aptamer technology to bridge precise diagnostics and targeted therapy, paving the way for innovative, personalized CVD interventions.)

## 1. Introduction

Cardiovascular diseases (CVDs) continue to be the leading cause of morbidity and mortality worldwide, accounting for approximately 32% of all global deaths, or an estimated 17.9 million lives lost annually [1,2]. The rising prevalence of CVDs is driven by factors such as aging populations, unhealthy lifestyles, and increasing rates of obesity, diabetes, and smoking [3]. Despite advancements in medical technology, current diagnostic and therapeutic strategies for CVDs face significant limitations. Traditional diagnostic methods, such as ELISA-based biomarker assays, often lack the sensitivity required for early detection, with limits of detection (LOD) for cardiac troponin I (cTnI) typically ranging from 1–10 ng/mL, which is insufficient for timely diagnosis of myocardial infarction [4]. Similarly, conventional anticoagulants and antiplatelet therapies suffer from systemic side effects, including increased bleeding risks, due to their non-specific mechanisms of action [5].

**Figure 1:**
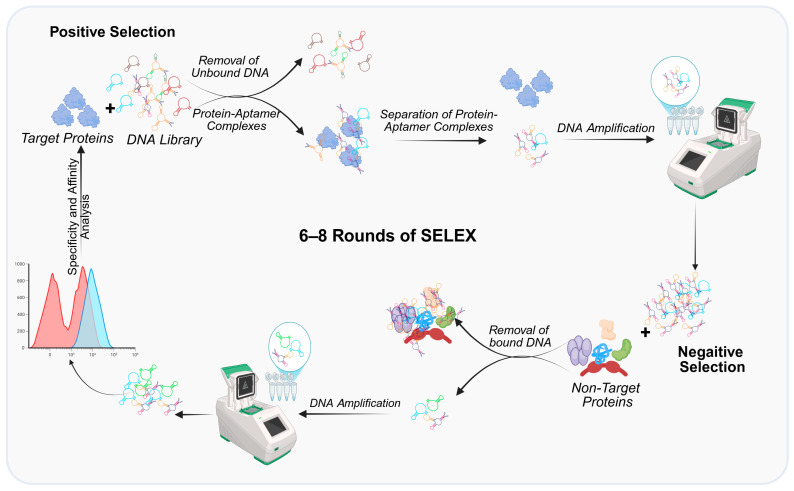
The Systematic Evolution of Ligands by Exponential Enrichment (SELEX) Process and Aptamer Architecture. Schematic representation of the SELEX process used for the selection of high-affinity DNA or RNA aptamers against cardiovascular targets. The workflow illustrates iterative rounds of target binding, partitioning, amplification, and enrichment, leading to the identification of highly specific aptamer sequences. Created in BioRender. Berezovski, M. (2026) https://BioRender.com/zykb2xk (accessed on 18 February 2026).

Aptamers, short single-stranded DNA or RNA molecules, have emerged as promising alternatives to overcome these challenges. Selected through the Systematic Evolution of Ligands by Exponential Enrichment (SELEX) process (Figure 1), aptamers exhibit high specificity and affinity for their targets, with dissociation constants (Kd) in the nanomolar range [6,7]. Unlike antibodies, aptamers offer several advantages, including lower production costs, ease of chemical modification, and reduced immunogenicity [8]. Their versatility enables applications across diagnostics, therapeutics, and drug delivery systems.

In diagnostics, aptamers have been developed to detect key cardiac biomarkers such as troponins (cTnI and cTnT), myoglobin, creatine kinase-MB (CK-MB), C-reactive protein (CRP), and natriuretic peptides (BNP and NT-proBNP). For instance, aptamers like TnIApt23 and Apt6 demonstrate high affinity for cTnI, with Kd values of 2.69 nM and 0.68 nM, respectively, enabling early and accurate detection of acute myocardial infarction [6,7]. Similarly, aptamer-based sensors for CRP and myoglobin have achieved attomolar sensitivity, outperforming traditional immunoassays [9,10].

Therapeutically, aptamers such as BT200 (targeting von Willebrand factor) and NU172 (targeting thrombin) show promise in clinical trials for their antithrombotic effects and rapid reversibility via antidote oligonucleotides [11,12]. Additionally, aptamer-mediated drug delivery systems, like Gint4.T and RNA-Apt30, enable precise modulation of cardiomyocyte function by targeting platelet-derived growth factor receptor beta (PDGFRβ) and phospholamban (PLN), respectively [13,14].

Recent advances in nanotechnology have further expanded the potential of aptamers. Integration with nanomaterials such as quantum dots, graphene, and DNA origami has led to the development of highly sensitive biosensors and targeted drug delivery platforms [15,16]. For example, aptamer-decorated liposomes and polymeric nanoparticles enhance the delivery of nucleic acids and therapeutic peptides to cardiac cells [17,18].

Despite these advancements, several challenges remain pertinent, including the need for broader clinical validation, optimization of aptamer stability in biological fluids, and overcoming PEG-related immunogenicity in therapeutic applications [19]. Nevertheless, the transformative potential of aptamers in uniting precise diagnostics and targeted therapy confirms their role as promising tools in the fight against cardiovascular diseases.

**Figure 2:**
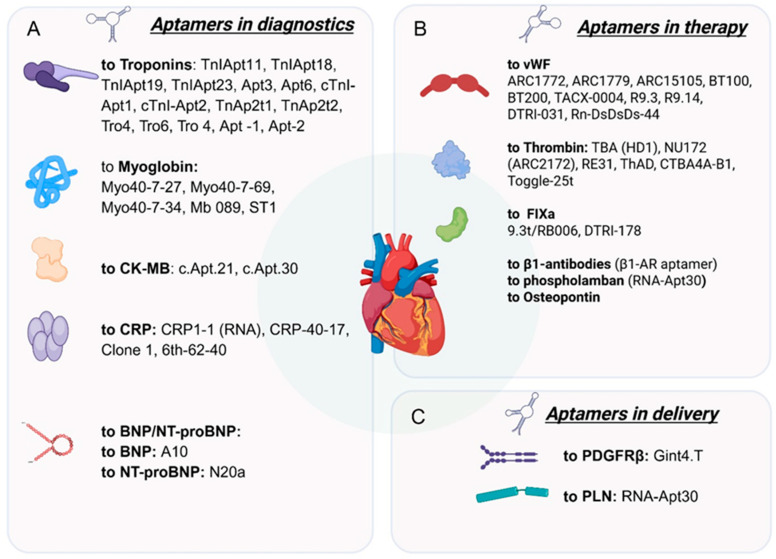
Dual Applications of Aptamers in Cardiovascular Medicine: Diagnostics and Targeted Therapy. This conceptual figure illustrates the versatility of aptamers in cardiology. (**A**) Diagnostic application: Aptamers immobilized on sensor surfaces (e.g., electrodes, chips) capture specific cardiac biomarkers—such as Cardiac Troponin I (cTnI), Myoglobin (Myo), C-Reactive Protein (CRP), and Brain Natriuretic Peptide (BNP)—from a blood sample, enabling detection and quantification. (**B**–**C**) Therapeutic and drug-delivery applications: (**B**) Aptamers can act as direct anticoagulants by inhibiting target proteins like Thrombin or von Willebrand Factor (vWF). (**C**) Alternatively, they serve as targeting ligands conjugated to drug carriers (e.g., nanoparticles, liposomes) or therapeutic payloads (peptides, small interfering RNA—siRNA) for cell-specific delivery to cardiomyocytes or vascular cells. Created in BioRender. Berezovski, M. (2026) https://BioRender.com/zykb2xk (accessed on 18 February 2026).

This review examines the current state of aptamer technology in cardiology, with a focus on diagnostic, therapeutic, and drug delivery applications, while also outlining key directions for future research and clinical translation (Figure 2). Specifically, for diagnostic purposes, aptamers are employed to create highly sensitive sensors capable of detecting key cardiac biomarkers.

## 2. Aptamers in Cardiovascular Disease Diagnostics

### 2.1. Troponins (cTnI and cTnT)

- Role: The “gold standard” for diagnosing acute myocardial infarction (AMI).
- Aptamers: Numerous DNA aptamers have been developed (e.g., TnIApt23, Apt6, Apt3) with high affinity (Kd in the nanomolar range).
- Applications: Used in electrochemical, optical, and luminescent biosensors, including in sandwich assay formats. Enable troponin detection in serum, blood, saliva, and urine with very low limits of detection (LOD).

One prominent area of research involves the identification and quantification of cardiac biomarkers using aptamers. Cardiac troponin is a biomarker for cardiac injury, with extensive evidence supporting its diagnostic and prognostic utility. Cardiac troponin, specifically troponin T and I, is regarded as the gold-standard biomarker in humans for detecting cardiac injury resulting from ischemic events and drug toxicity. This biomarker’s sensitivity and specificity have been demonstrated across various studies, establishing its fundamental role in clinical assessment [20]. Aptamers to troponins I and T are presented in Table 1.

The aptamer TnIApt23 selection and characterization have been documented across multiple studies, highlighting its high affinity and potential diagnostic utility.

Initially, TnIApt23 was identified among four aptamer sequences—TnIApt23, TnIApt19, TnIApt18, and TnIApt11—through a selection process aimed at isolating DNA aptamers against human cardiac troponin I [24]. Among these, TnIApt23 demonstrated the highest affinity for the target protein, with a nanomolar range dissociation constant of approximately 2.69 nM, indicating a strong and specific interaction [6]. This high affinity was further confirmed in subsequent testing, where TnIApt23 consistently exhibited superior binding characteristics compared to other aptamers in the same group [8].

Cen et al. highlighted that Apt3 and Apt6 exhibit strong binding affinity and specificity, enabling their use in a dual-aptamer sandwich ELONA method that offers a broad detection range for cTnI in serum samples [7]. This approach underscores the potential of these aptamers in clinical diagnostics, particularly for early and accurate detection of AMI. Similarly, the selection process utilizing magnetic bead-SELEX technology further confirmed the high affinity of Apt3 and Apt6 for cTnI, emphasizing their suitability as analytical tools [7].

The structural and functional advantages of these aptamers have also been explored in the context of biosensor development. DNAzyme-based biosensors incorporating Apt3 have been proposed for sensitive and accurate cTnI detection, demonstrating the versatility of these aptamers in different biosensing platforms [25].

A dual signal amplified electrochemical aptasensor has been constructed utilizing Apt1 immobilized on a gold electrode via self-assembled Au–S bonds, enabling specific capture of cTnI [26]. This approach enhances detection sensitivity by integrating amplification strategies, demonstrating the potential of Apt1 in electrochemical sensing platforms. The application of aptamers in enzyme-linked hybrid-sandwich assays has been explored, emphasizing the specificity of Apt1 for cTnI detection. The assay’s design involves two anti-cTnI aptamers, including Apt1, to improve selectivity and sensitivity. Complementing these developments, a highly sensitive electrochemical biosensor was reported where Apt1 captures cTnI on the electrode surface, followed by the addition of Apt2 to form a sandwich complex, thereby enhancing detection accuracy [26].

A sandwich bioluminescent assay based on TnAp10 and TnAp2t3, which are related aptamers, has been developed to detect cTnI concentrations ranging from 0.04 to 3 nM [18].

The Tro4 aptamer, which was initially developed for cTnI [21], has also been utilized in electrochemical sensing of cTnT [22]. Although selectivity tests were conducted for both aptasensors, cTnI was not evaluated as a potential interferent. Meanwhile, other studies have examined Tro4′s specificity toward cTnT, with cTnI-targeting aptasensors showing no cross-reactivity to cTnT [27]. However, the existing data on Tro4′s specificity for cTnT remains inconsistent, highlighting the need for thorough validation of aptamer selectivity against both intended targets and relevant cross-reactants.

Additionally, two DNA aptamers (Apt1 and Apt2) have been employed in an ELONA sandwich assay for cTnT [23]. While this assay’s limit of detection (LOD) and dynamic range were insufficient for clinical cTnT measurement, it demonstrated efficient recovery of the analyte from undiluted serum [23]. The performance of Apt1 and Apt2 could be enhanced through optimized biosensor design. For instance, an electrochemical Apt1-based sensor achieved an LOD of 1.7 pg/mL in 10-fold diluted serum—sufficient for practical applications—while exhibiting no cross-reactivity with cTnI or myoglobin [28].

A wide range of platforms exists for detecting troponin using aptamer-based sensors, encompassing various types such as optical methods (including luminescence, fluorescence, surface plasmon resonance, surface-enhanced Raman scattering, and colorimetry), along with electrochemical biosensors (differential pulse voltammetry, cyclic voltammetry, square wave voltammetry, electrochemical impedance spectroscopy, field-effect transistors) [4,29,30]. These developed aptasensors generally exhibit outstanding performance features. Established aptamers are suitable not only for lab-based tests requiring instrumentation but also for point-of-care portable gadgets [31]. They may further enhance their effectiveness when used in combination within a sandwich-like configuration [32]. It has already been demonstrated that these aptamers can identify cardiac troponin I from diverse biological samples like saliva, urine, complete blood, and even blood serum [33,34].

### 2.2. Myoglobin

- Role: An early but non-specific marker for AMI and rhabdomyolysis.
- Aptamers: The most studied is aptamer Myo40-7-27 (Kd = 4.93 nM).
- Applications: Electrochemical and optical sensors. A key limitation is the lack of dual-aptamer sandwich assays, which restricts sensitivity.

Myoglobin, a small heme-containing protein (16.8 kDa) predominantly found in cardiac and skeletal muscle tissue, plays a critical role in oxygen storage and transport. Due to its rapid release into the bloodstream following muscle injury, myoglobin serves as an early biomarker for acute myocardial infarction (AMI) and rhabdomyolysis. Serum myoglobin levels serve as an early but nonspecific biomarker for acute myocardial infarction, typically rising within 1 h and peaking at 4–12 h post-injury. Normal blood levels range from 6–85 ng/mL, with AMI diagnostic thresholds of 70–200 ng/mL, while concentrations exceeding 5000 ng/mL are strongly suggestive of rhabdomyolysis resulting from muscle trauma, infections, or toxic exposures. It can also be detected in saliva (correlating with serum levels) and urine (where it indicates renal or muscle damage rather than cardiac injury). Despite its rapid release kinetics making myoglobin useful for initial assessment, its diagnostic utility in cardiac events remains limited without concurrent measurement of more specific markers like cardiac troponins and CK-MB due to shared expression in skeletal muscle tissue [22,35,36].

Several DNA aptamers have been developed for myoglobin detection. The most extensively studied is Myo40-7-27 (5′-CCCTCCTTTCCTTCGACGTAGATCTGCTGCGTTGTTCCGA-3′), a 40-nt aptamer with a dissociation constant (Kd) of 4.93 nM, as determined by surface plasmon resonance (SPR) [10,37]. This aptamer has been widely employed in electrochemical and optical biosensors, including sandwich assays paired with antibodies [38]. Two related 40-nt aptamers, Myo40-7-69 (5′-CGAGTACTTCTTTGCTAGTTCGCGAGATACGTTGGCTAGG-3′) and Myo40-7-34 (5′-ACGCACAATTCCTTGTCCAATTAGGAAATTCTACGCGGAT-3′), exhibit slightly lower affinities (Kd = 6.38 nM and 5.58 nM, respectively) but have seen limited application in sensing platforms [39]. Another aptamer, Mb 089 (72 nt; sequence: 5′-ATCCGTCACACCTGCTCTTAATTACAGGCAGTTCCACTTAGACAGACACACGAATGGTGTTGGCTCCCGTAT-3′), was used in an electrochemical sensor with a detection limit (LOD) of 2.1 pg/mL in diluted serum [40]. The ST1 aptamer (sequence undisclosed) enabled ultrasensitive surface-enhanced Raman spectroscopy (SERS) detection (LOD = 10 fg/mL) [41]. Additionally, a 78-nt DNA aptamer (5′-ATCCAGAGTGACGCAGCACAACGTGCAAATTATACCTGTTTTCCCCTTTTCCTACAAGTGCTATGGACACGGCTTAGT-3′) demonstrated picomolar affinity and was applied in serum-based electrochemical assays without cross-reactivity to hemoglobin or albumin [42].

Despite these advances, challenges remain. No dual-aptamer sandwich assays have been reported, limiting sensitivity improvements achievable through simultaneous binding at multiple epitopes. Most studies focus on serum, with only a few exploring saliva or urine detection. Future work should prioritize clinical validation in non-invasive samples and the development of standardized, high-throughput aptamer-based assays to compete with conventional immunoassays.

### 2.3. Creatine Kinase-MB (CK-MB)

- Role: A historical marker of myocardial injury.
- Aptamers: c.Apt.21 (Kd = 0.81 nM) and c.Apt.30 (Kd = 24.04 nM).
- Applications: Used in microfluidic platforms and fluorescent lateral flow assays for rapid point-of-care testing.

Creatine kinase (CK) is a crucial enzyme involved in cellular energy metabolism, particularly in tissues with high energy demands like cardiac and skeletal muscle. Creatine kinase-MB (CK-MB) isoenzyme has historically served as a cardinal biomarker for acute myocardial injury, especially in the context of myocardial infarction. CK-MB begins to elevate within hours of cardiac injury, reaching peak levels 6–12 h post-onset and returning to baseline within 24–48 h [43]. While conventional detection methods rely on antibody-based immunoassays or enzymatic activity measurements, recent advances in aptamer technology offer promising alternatives with potential advantages in cost, stability, and versatility. Two DNA aptamers have been specifically developed for CK-MB detection. The first, designated c.Apt.21, is a 45-nucleotide sequence (GGGGGGTGGGTGGGGGATCTCGGAGGATGCTTTTAGGGGGTTGGG) demonstrating high affinity with a dissociation constant of 0.81 nM. This aptamer was successfully incorporated into an innovative microfluidic platform using DNA hydrogel technology, enabling quantitative measurements with a smartphone camera and achieving a detection limit of 0.027 nM across a wide linear range [44,45]. The second aptamer, c.Apt.30 (CATTGAGAGGGGGTGGCCGTAGTCAGGTGGGTGGGGGTTTGAG), while showing slightly lower affinity (Kd = 24.04 nM), proved effective in a fluorescent lateral flow assay format with sensitivity sufficient for clinical applications. These aptamer-based approaches represent significant progress in biosensor development, particularly for point-of-care testing scenarios where rapid results and simplified instrumentation are advantageous. However, several challenges remain to be addressed, including the need for improved specificity to distinguish CK-MB from other isoforms and the development of dual-aptamer sandwich assays for enhanced sensitivity. Additionally, while preliminary studies have explored CK-MB detection in saliva as a non-invasive alternative, further validation is required to establish reliable correlations with serum levels and clinical outcomes [46]. Future research directions should focus on integrating CK-MB aptasensors with other cardiac biomarker detection systems to create comprehensive diagnostic panels and optimizing aptamer performance in complex biological matrices. The evolution of these technologies may eventually lead to more accessible and cost-effective tools for cardiovascular disease monitoring, particularly in resource-limited settings where traditional laboratory methods are impractical.

### 2.4. C-Reactive Protein (CRP)

- Role: A marker of systemic inflammation and cardiovascular risk.
- Aptamers: Both DNA (e.g., CRP-40-17, Clone 1) and high-affinity RNA aptamers (CRP1-1, Kd = 2.25 nM) have been developed.
- Applications: Highly sensitive detection using surface plasmon resonance (SPR), electrochemical, and colorimetric methods. Combined “aptamer-antibody” sandwich assays are particularly effective.

The significance of CRP, particularly high-sensitivity CRP (Hs-CRP), in the pathogenesis of CVD has garnered considerable attention in recent research. CRP, a homopentameric protein produced in the liver, is recognized as a pivotal biomarker of systemic inflammation, which plays a crucial role in the development and progression of atherosclerosis, a primary underlying cause of many cardiovascular conditions [47].

The predictive value of CRP in cardiovascular events has been substantiated through large-scale analyses. Ridker P.M. et al. evaluated the relative importance of CRP alongside low-density lipoprotein cholesterol (LDL-C) in predicting adverse cardiovascular outcomes among patients on statin therapy. Their findings suggest that CRP remains a significant independent predictor of cardiovascular events, reinforcing its role in risk stratification [48]. While not cardiac-specific, CRP elevations strongly correlate with atherosclerotic inflammation, with concentrations <1 μg/mL indicating low risk, 1–3 μg/mL intermediate risk, and >3 μg/mL high cardiovascular risk. During acute myocardial infarction, CRP levels typically exceed 10 μg/mL, reaching up to 200 μg/mL in severe inflammatory states [49].

In addition to its diagnostic and prognostic utility, CRP’s role in the broader context of inflammation-related cardiovascular pathogenesis is supported by its association with other inflammatory markers and clinical outcomes. There is a relationship between inflammatory markers, including CRP, and microvascular parameters, further illustrating the systemic impact of inflammation on cardiovascular health [50]. The protein’s detection in urine shows better correlation with serum levels than salivary measurements, making it potentially valuable for non-invasive monitoring [51].

Multiple DNA and RNA aptamers targeting CRP have been developed through various selection techniques. The RNA aptamer CRP1-1 (GGGCGAAUUCGGGACUUCGAUCCGUAGUACCCACCAGGCAUACACCAGCACGCGGAGCCAAAGAAAAAUAGUAAACUAGCACUCAGUGCUCGUAUGCGGAAGCU) demonstrates exceptional affinity with Kd = 2.25 nM [52]. DNA aptamers include CRP-40-17 (CCCCCGCGGGTCGGCTTGCCGTTCCGTTCGGCGCTTCCCC; Kd = 16.2 nM) selected via GO-SELEX [53], and Clone 1 (GGCAGGAAGACAAACACGATGGGGGGGTATGATTTGATGTGGTTGTTGCATGATCGTGGTCTGTGGTGCTGT; Kd = 3.51 nM) identified through microfluidic SELEX [54]. Particularly noteworthy is the 20-mer DNA sequence (GGGCCTCCGGTTCATGCCGC) that achieved remarkable sensitivity in surface plasmon resonance assays when paired with antibodies [9]. Aptamer-based CRP detection has been implemented across diverse platforms, including electrochemical, colorimetric, and surface-enhanced Raman spectroscopy (SERS) systems, with some achieving attomolar sensitivity through signal amplification strategies. A critical advancement involves the development of sandwich-type assays combining aptamers with antibodies, as demonstrated in an SPR-based approach using aptamer 6th-62-40 (CGAAGGGGATTCGAGGGGTGATTGCGTGCTCCATTTGGTG), which improved detection limits 100-fold compared to direct aptamer sensing [55]. While most validation studies have focused on serum samples, successful CRP quantification has also been reported in urine and whole blood, highlighting the technology’s potential for point-of-care applications [56,57]. Current limitations include the scarcity of validated dual-aptamer pairs for sandwich assays and insufficient clinical data comparing aptamer-based results with high-sensitivity CRP immunoassays in diverse patient populations [9,55].

### 2.5. Natriuretic Peptides (BNP and NT-proBNP)

- Role: Key markers for diagnosing and prognosticating heart failure.
- Aptamers: A10 (for BNP, Kd = 12 nM) and N20a (for NT-proBNP, Kd = 2.89 nM).
- Applications: Used in microfluidic systems and electrochemical sensors for highly sensitive detection. Challenges include the low concentration and rapid in vivo degradation of BNP.

The natriuretic peptide system plays a crucial role in cardiovascular homeostasis, with BNP and its inactive N-terminal fragment (NT-proBNP) serving as clinically established biomarkers for heart failure diagnosis and prognosis. These peptides are released from cardiomyocytes in response to ventricular wall stress, with BNP exhibiting biological activity through vasodilation and natriuresis, while NT-proBNP serves as a more stable surrogate marker due to its longer plasma half-life (1–2 h vs. 20 min for BNP). The clinical utility of these markers is complicated by BNP’s rapid degradation in circulation and the presence of immunoreactive but biologically inactive fragments that can interfere with traditional antibody-based assays. This has spurred interest in aptamer-based detection methods that can potentially overcome these limitations through enhanced specificity and stability.

Several DNA aptamers have been developed for BNP detection, beginning with the 55-mer sequence (TAAACGCTCAAAGGACAGAGGGTGCGTAGGAAGGGTATTCGACAGGAGGCTCACA) identified in 2009, which demonstrated utility in surface plasmon resonance (SPR) and electrochemical platforms when combined with antibodies. Subsequent selections yielded the high-affinity A10 aptamer (GGCGATTCGTGATCTCTGCTCTCGGTTTCGCGTTCGTTCG; Kd = 12 nM), which has been incorporated into photoelectrochemical and differential pulse voltammetric sensors, achieving remarkable sensitivity (LOD = 0.14 pg/mL) [58]. Other candidate sequences from magnetic bead-based SELEX (2F, 6R, 14bF, 25cF) showed promise for sandwich assays, though cross-reactivity with serum proteins remains a challenge. For NT-proBNP, the N20a aptamer (GGCAGGAAGACAAACAGGTCGTAGTGGAAACTGTCCACCGTAGACCGGTTATCTAGTGGTCTGTGGTGCTGT; Kd = 2.89 nM) has enabled microfluidic detection with clinical correlation to established immunoassays, while also functioning in amperometric sensor configurations [59].

The development of aptamer-based detection systems for natriuretic peptides faces unique challenges. BNP’s low physiological concentrations (normal: ~20 pg/mL) and rapid degradation necessitate exceptionally sensitive and robust detection platforms [60]. The A10 aptamer has shown particular promise in this regard, with demonstrated functionality in serum matrices and wide dynamic ranges spanning 1 pg/mL to 0.1 μg/mL in optimized assays [61]. For NT-proBNP, the N20a aptamer’s performance in automated microfluidic systems (LOD = 1.53 pg/mL, 86–97% recovery) suggests potential for point-of-care applications, though clinical validation studies remain limited compared to conventional immunoassays [59].

Current limitations in the field include the absence of fully aptamer-based sandwich assays for NT-proBNP and insufficient clinical data comparing aptamer and antibody performance across diverse patient populations. The structural similarity between BNP, NT-proBNP, and related natriuretic peptides presents ongoing specificity challenges that may require negative selection strategies during aptamer development.

Aptamer-based biosensors have demonstrated remarkable sensitivity for cardiac biomarker detection, often outperforming conventional immunoassays. Electrochemical and optical platforms enable detection of troponins, CRP, and natriuretic peptides at clinically relevant concentrations. However, clinical translation remains limited by the lack of standardized validation in patient cohorts, scarce development of dual-aptamer sandwich assays, and insufficient testing in complex biological matrices. Future efforts should focus on multiplexed microfluidic platforms and rigorous clinical validation to establish point-of-care utility.

## 3. Therapeutic Aptamers for Antithrombotic Therapy

The hemostatic system is a complex entity comprising coagulation, anticoagulation, and fibrinolytic systems, which collectively maintain blood in a fluid state, halt bleeding upon vascular injury, and dissolve thrombi. Due to the system’s multicomponent nature, the development of novel diagnostic methods and modern therapeutics with high selectivity is of considerable relevance. One promising solution involves the engineering of molecular recognition elements capable of selectively inhibiting one arm of the hemostatic system and/or activating another. Among the most promising anticoagulants, offering minimal side effects and pronounced biological activity, are aptamers, which have recently garnered significant scientific interest [62].

### 3.1. Von Willebrand Factor (vWF) Aptamers

- Target: vWF, a key protein in platelet adhesion.
- Aptamers:.ARC1772/ARC1779: The first aptamers to show efficacy in clinical trials.
- BT200: An improved pegylated aptamer with prolonged action, undergoing preclinical and clinical investigation. Has demonstrated a cardioprotective effect in ischemia–reperfusion models.
- TACX-0004: Incorporates artificial hydrophobic nucleotides to enhance binding affinity.
- Advantage: The availability of antidotes (e.g., BT101) allows for rapid reversal of the anticoagulant effect.

Von Willebrand factor (vWF) is a large multimeric glycoprotein that plays a pivotal role in hemostasis by mediating platelet adhesion to the subendothelial collagen matrix and promoting platelet aggregation, particularly under high shear stress conditions found in stenotic arteries and microcirculation. The A1 domain of vWF interacts with the platelet receptor glycoprotein Ib (GPIb), initiating thrombus formation, making vWF a critical target for antithrombotic therapies [63]. Dysregulation of vWF is implicated in thrombotic disorders such as thrombotic thrombocytopenic purpura (TTP), stroke, and myocardial infarction, underscoring its relevance as a therapeutic biomarker [11].

Aptamers targeting vWF offer a promising alternative to conventional antiplatelet agents due to their high specificity, reversible action, and reduced risk of immunogenicity. The first vWF-targeting aptamer, ARC1772 (sequence: *5′-GGTTGGTGTGGTTGG-3′*), was a DNA aptamer binding the GPIb-binding A1 domain [64]. Its optimized pegylated variant, ARC1779, demonstrated potent inhibition of vWF-mediated platelet aggregation (Kd ≈ 2 nM) and was evaluated in clinical trials for carotid endarterectomy, where it reduced thromboembolic events but was associated with bleeding risks and injection-site reactions [65]. Another aptamer, ARC15105, exhibited efficacy comparable to the monoclonal antibody abciximab in inhibiting platelet adhesion [66]. Further modifications, such as the addition of stabilizing nucleotides, yielded BT100, which was subsequently pegylated to create BT200 (sequence not fully disclosed but derived from BT100). BT200 showed robust antithrombotic activity in cynomolgus monkeys and is under investigation for secondary prevention of arterial thrombosis [11,64]. Recent studies in a murine model of myocardial ischemia–reperfusion injury have demonstrated that the BT200 aptamer not only inhibits the A1 domain activity of vWF and prolongs bleeding time, but also significantly reduces infarct size, improves cardiac function, and attenuates microvascular obstruction and cardiomyocyte apoptosis [67]. To address bleeding risks, a complementary antidote aptamer, BT101, was developed to rapidly reverse BT200′s effects [11].

Structural advancements include the aptamer TACX-0004 (sequence: *5′-Ds-Ds-Ds-GGTTGGTGTGGTTGG-Ds-Ds-Ds-3′*, where Ds denotes a hydrophobic artificial base), which incorporates a synthetic hydrophobic base to enhance binding affinity (Kd = 2.2 ± 0.9 nM) [68]. Comparative studies revealed TACX-0004′s superior inhibition of vWF A1 compared to ARC1779 and caplacizumab, attributed to its unique interaction with residue F1366 in the A1 domain. Other aptamers, such as R9.3 and R9.14, demonstrated complete inhibition of platelet plug formation at concentrations > 40 nM, with activity reversible by the oligonucleotide antidote AO6 [69]. Similarly, the RNA aptamer DTRI-031 (sequence: *5′-GGGCGAUUGUUGUGUGUGUGUGUGUGUGUGUGUCCCC-3′*) and its antidote showed efficacy in murine thrombosis models [70], while Rn-DsDsDs-44 (sequence: *5′-Rn-Ds-Ds-Ds-GGTTGGTGTGGTTGG-Ds-Ds-Ds-Rn-3′*), stabilized by mini-hairpin loops (Rn), exhibited high vWF affinity [71].

Clinical trials of vWF aptamers highlight both potential and challenges. ARC1779 reduced embolic signals in carotid endarterectomy patients but faced limitations due to bleeding and PEG-related immunogenicity [65]. BT200′s preclinical success positions it as a candidate for stroke and myocardial infarction prevention, though its clinical translation requires further validation [11]. The development of antidote-controllable aptamers, such as those paired with AO6 or BT101, addresses safety concerns but necessitates rigorous evaluation of reversal kinetics and long-term effects [69,70].

vWF-targeting aptamers represent a versatile tool for antithrombotic therapy, with structural modifications enhancing stability and efficacy. While clinical studies underscore their potential, challenges such as bleeding risks and immunogenicity must be resolved to advance their therapeutic application. Future directions include optimizing pharmacokinetics and expanding clinical trials to validate their utility in acute and chronic thrombotic conditions.

### 3.2. Thrombin Aptamers

- Target: Thrombin, the central enzyme in the coagulation cascade.
- Aptamers: TBA (HD1): The classic 15-mer DNA aptamer that forms a G-quadruplex structure.
- NU172: A more stable 26-mer aptamer that advanced to clinical trials.
- RE31: An engineered aptamer with improved properties.
- Toggle-25t RNA aptamer: The aptamer binds to the thrombin region known as exosite II.
- Development: Bivalent and circular aptamers are being created to enhance stability and efficacy.

Thrombin (activated coagulation factor II, Factor IIa, FIIa) is a key serine protease in the coagulation cascade that converts fibrinogen to fibrin and activates platelets, making it a prime target for antithrombotic therapies [72]. The development of thrombin-binding aptamers represents a significant advancement in anticoagulant treatment due to their high specificity, reversible action, and reduced bleeding risks compared to traditional therapies like heparin. One of the most studied thrombin aptamers is the thrombin-binding aptamer (TBA, also known as HD1) with the sequence 5′-GGTTGGTGTGGTTGG-3′. This 15-mer DNA aptamer folds into a stable G-quadruplex structure with two G-tetrads stabilized by potassium ions and three loops (two TT loops and one TGT loop) that enable its high-affinity binding (Kd ≈ 4.5–8.2 nM) to thrombin’s exosite I, effectively inhibiting fibrin clot formation. Despite its promising mechanism, TBA’s clinical application has been limited by rapid clearance from circulation, prompting the development of modified variants with improved pharmacokinetic properties [73,74].

A notable advancement came with NU172 (ARC2172, sequence: 5′-CGCCTAGGTTGGGTAGGGTGGCCG-3′), a 26-mer DNA aptamer that combines G-quadruplex and duplex domains to achieve stronger thrombin inhibition (Kd ≈ 12 nM) and better stability. This aptamer progressed to Phase IIa clinical trials for use in cardiopulmonary bypass procedures, demonstrating rapid anticoagulation with potentially fewer bleeding complications than heparin, though further development has stalled [12]. Another engineered aptamer, RE31 (sequence: 5′-GTGACGTAGGTTGGTGTGGGCTTGGCGCGTCAC-3′), incorporates an extended duplex region that enhances its binding to thrombin’s exosite I, showing superior antithrombotic effects in preclinical rat models compared to TBA. The pharmacokinetics of RE31 were further improved by PEGylation, highlighting how strategic modifications can optimize aptamer performance [75,76].

**Figure 3:**
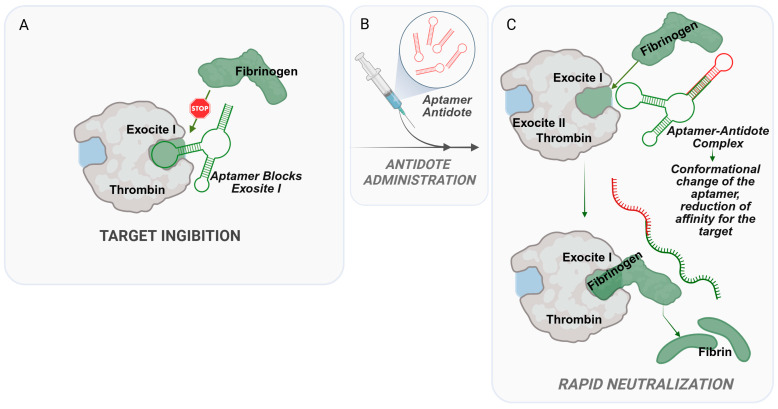
The Principle of Reversible Action of Therapeutic Aptamers: Controllable. (**A**) Target Inhibition. A structured DNA aptamer (green) binds with high affinity to exosite I on the coagulation factor thrombin (gray), sterically blocking its active site and inhibiting procoagulant activity. (**B**) Antidote Administration. To reverse the effect, a complementary antisense oligonucleotide “antidote” is administered intravenously. (**C**) Rapid Neutralization. The antidote hybridizes with the aptamer, inducing a conformational change that unfolds the aptamer and releases thrombin, restoring hemostatic function within minutes. Created in BioRender. Berezovski, M. (2026) https://BioRender.com/zykb2xk (accessed on 18 February 2026).

Recent innovations include bivalent aptamers like ThAD that simultaneously target thrombin’s exosite I and II for enhanced inhibition, and circular aptamers such as CTBA4A-B1, which demonstrate remarkable nuclease resistance with a half-life of about 8 h [77,78]. Sullenger and colleagues demonstrated that the Toggle-25t RNA aptamer interacts with thrombin at its heparin-binding site and exhibits an extensive molecular surface complementary to the protein. Protein recognition is mediated by the stacking of single-stranded adenine bases within the aptamer’s tertiary core with arginine side chains [79]. These developments address key challenges in aptamer therapeutics, particularly their susceptibility to degradation and rapid clearance. While thrombin aptamers offer significant advantages over conventional anticoagulants [78], including the potential for rapid reversal with complementary oligonucleotide antidotes (Figure 3), their clinical translation requires further optimization of dosing regimens and larger-scale trials to validate their efficacy and safety in conditions like acute coronary syndromes and post-surgical thrombosis.

### 3.3. Factor IXa Aptamers

- Target: Factor IXa, a key enzyme in the intrinsic coagulation pathway.
- The REG1 System: Consisted of the aptamer RB006 (pegnivacogin) and its antidote RB007. It was the first aptamer-antidote pair to be tested in humans.
- Challenge: Clinical development was halted due to hypersensitivity reactions linked to pre-existing anti-polyethylene glycol (PEG) antibodies.

Factor IXa (FIXa) plays a crucial role in the intrinsic pathway of blood coagulation, where it activates Factor X in complex with Factor VIIIa, making it an attractive target for anticoagulant therapy. The development of FIXa-targeting aptamers has emerged as a promising strategy due to their high specificity and the potential for rapid reversal of anticoagulant effects. One of the most extensively studied aptamers in this category is the 9.3t aptamer (sequence: 5′-GGGAGGACGAUGCGGACCGAAAAGGUUCCUCCC-3′), which binds to an extended substrate-binding site on FIXa, effectively inhibiting FIXa-mediated activation of Factor X without interfering with the formation of the FVIIIa/FIXa complex. This RNA aptamer demonstrates remarkable specificity, as it does not affect other coagulation factors, highlighting its potential as a targeted anticoagulant [80].

Further advancements led to the development of the REG1 system, which consists of the FIXa-binding aptamer RB006 (sequence: 5′-GGGAGGACGAUGCGGACCGAAAAGGUUCCUCCC-3′, a slightly modified version of 9.3t) and its complementary antidote RB007. This system represents the first aptamer-antidote pair to enter clinical trials, demonstrating the feasibility of controlled anticoagulation in humans. Phase I studies in healthy volunteers and patients with stable coronary artery disease showed that REG1 provided predictable and reproducible anticoagulation that could be immediately reversed by administration of RB007, offering a significant advantage over conventional anticoagulants [81]. The antidote RB007 works by forming a double-stranded RNA hybrid with RB006, causing a conformational change that releases FIXa and restores normal coagulation within minutes. This rapid reversibility is particularly valuable in clinical settings where uncontrolled bleeding may occur, such as during surgery or trauma. But the PEGylated aptamer RB006 (pegnivacogin), despite demonstrating promising anticoagulant activity through Factor IXa inhibition, faced clinical failure in Phase III trials due to severe hypersensitivity reactions linked to pre-existing anti-PEG antibodies in patients. This case highlighted the critical immunogenicity challenges of PEGylated therapeutics, ultimately leading to the termination of the REG1 system development and underscoring the need for alternative, non-immunogenic delivery strategies in aptamer design [19].

Another notable FIXa aptamer, DTRI-178, was derived from 9.3t and evaluated in preclinical studies. In porcine models, DTRI-178 showed comparable thromboprophylactic efficacy to unfractionated heparin but with significantly reduced bleeding at the surgical site, suggesting a better safety profile [82]. The reduced bleeding risk may be attributed to the aptamer’s specific mechanism of action, which selectively targets FIXa without affecting other components of the coagulation cascade. This specificity contrasts with heparin, which exerts a broader anticoagulant effect through interactions with multiple coagulation factors. The development of these FIXa aptamers has been facilitated by modifications such as 2′-fluoropyrimidine incorporation, which enhances nuclease resistance and prolongs circulation time without compromising binding affinity.

Despite these promising developments, challenges remain in the clinical translation of FIXa aptamers. The REG1 system, while demonstrating proof-of-concept for controllable anticoagulation, revealed limitations related to variable patient responses and the potential for immunogenicity against the PEG component used in some formulations [83].

### 3.4. Clinical Translation of Antithrombotic Aptamers

The promising preclinical profile of aptamers, characterized by high specificity, controllability, and rapid reversibility, has motivated their translation into clinical trials for various cardiovascular indications. Table 2 provides a comprehensive overview of the clinical development status of key antithrombotic aptamers discussed in this review. This summary includes their molecular targets, clinical trial identifiers, phases, and key outcomes. It is noteworthy that the clinical landscape reveals both encouraging successes, demonstrating proof-of-concept for aptamer-antidote pairs, and valuable setbacks, such as the hypersensitivity reactions observed with the PEGylated aptamer in the REG1 system, which highlight critical challenges for future aptamer design and formulation.

Antithrombotic aptamers offer unique advantages over conventional anticoagulants, including high specificity and rapid reversibility via antidote oligonucleotides. Clinical candidates such as BT200 (anti-vWF) and NU172 (anti-thrombin) have shown promise, while the REG1 system (anti-FIXa) provided critical lessons about PEG-related immunogenicity. Despite these setbacks, the field continues to evolve with improved chemistries and smarter inhibitor designs. Future success requires non-immunogenic stabilization strategies, optimized dosing regimens, and adequately powered clinical trials in targeted patient populations.

## 4. Aptamers for the Diagnosis and Treatment of Heart Failure

Heart failure is a pathophysiological syndrome characterized by an impairment of the cardiac pumping function, leading to a disparity between the body’s hemodynamic demands and the heart’s capacity. Aptamers represent a promising platform for both diagnostic [84] and therapeutic applications in the context of heart failure [85].

### 4.1. Biomarker Detection of Heart Failure

Aptamers can be used in assays to detect specific biomarkers in a patient’s blood, which can aid in the diagnosis of heart failure [86]. An aptamer targeting soluble ST2 (sST2) has shown the ability to differentiate heart failure patients from healthy volunteers [87,88].

### 4.2. Therapeutic Agents

Aptamers are being developed as a new class of drugs to treat heart failure [89,90]. Aptamers can be engineered to target specific molecules implicated in the pathogenesis of heart failure. Specifically, the β1-AR aptamer, which neutralizes autoantibodies against the β1-adrenergic receptor (β1-AR), could serve as an adjunct to standard heart failure therapies [86]. An aptamer targeting phospholamban can attenuate its inhibitory effect on SERCA2a, thereby improving calcium handling in cardiomyocytes. This approach is currently being explored in the development of aptamers that are also capable of being delivered into the intracellular compartment of cardiac cells [89]. An aptamer targeting Osteopontin (OPN) has shown potential in preventing and reversing pressure-overload-induced heart failure in animal models. The authors utilized a mouse model of pressure overload. Administration of the aptamer during the surgical procedure prevented cardiomyocyte hypertrophy and cardiac fibrosis, inhibited OPN signaling pathways, and averted the onset of heart failure. Notably, when administered two months post-surgery, the aptamer treatment reversed established heart failure, fibrosis, and myocyte hypertrophy. The authors concluded that modulation of OPN signaling pathways via the aptamer represents a novel and effective strategy for preventing cardiac hypertrophy and fibrosis, improving cardiac function, and reversing pressure overload-induced heart failure [85]. Aptamers can be engineered to block pathological signaling pathways involved in heart failure [91]. Aptamers can be used to deliver drugs or other therapeutic agents directly to the heart or to specific cells involved in the disease process. They can be conjugated with other molecules, like siRNAs, to enhance their therapeutic effects and enable targeted delivery. Research is exploring aptamer-drug conjugates for applications like drug-eluting stents [8].

## 5. Aptamers for Targeted Drug Delivery

### 5.1. Gint4.T (Against PDGFRβ)

**Figure 4:**
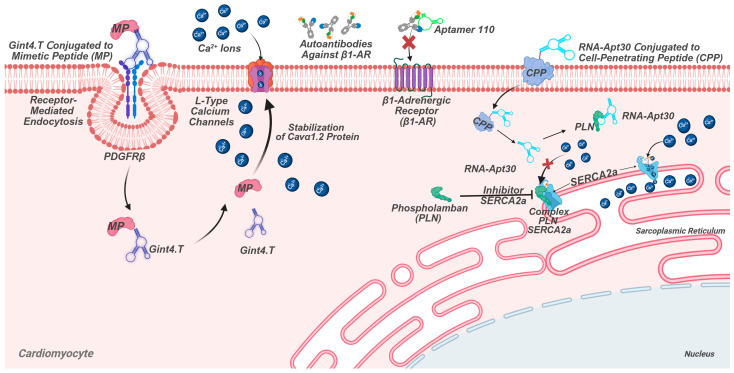
Aptamer-Based Interventions for Heart Failure Therapy. Three-level strategy for targeted modulation of cardiomyocyte function. A schematic cross-section of a cardiomyocyte illustrates three distinct levels of aptamer intervention: Extracellular Neutralization: A β1-AR-specific DNA aptamer binds pathological autoantibodies, blocking their chronic activation of the β1-adrenergic receptor (β1-AR). Created in BioRender. Berezovski, M. (2026) https://BioRender.com/zykb2xk (accessed on 18 February 2026).

- Target: The PDGFRβ receptor, expressed on cardiomyocytes.
- Application: The RNA aptamer Gint4.T is used as a “vehicle” to deliver therapeutic peptides directly to heart cells. It has been shown to effectively restore the function of calcium channels in cardiomyocytes (Figure 4).

Platelet-derived growth factor receptor beta (PDGFRβ) is a key tyrosine kinase receptor that plays a crucial role in cellular processes such as proliferation, migration, and survival. In the cardiovascular system, PDGFRβ is expressed in cardiomyocytes, smooth muscle cells, and fibroblasts, making it an important target for cardiac-specific therapies [92]. Given its selective expression, PDGFRβ has attracted attention as a biomarker for targeted drug delivery in cardiology [13]. Aptamers, due to their high binding affinity and specificity, have emerged as promising tools for such applications. Among them, the RNA aptamer Gint4.T has been extensively studied for its ability to bind PDGFRβ and facilitate intracellular delivery of therapeutic agents [93].

Gint4.T is a 53-nucleotide RNA molecule chemically modified with 2′-fluoro pyrimidines to enhance its stability against enzymatic degradation (original structure of the aptamer 5′-UUGUGUGGGGCAUCCAGUAAAUGCAAUUCGACA-3′) [13,93]. The aptamer exhibits high affinity for PDGFRβ, enabling efficient receptor-mediated internalization. A notable feature of Gint4.T is its adaptability for conjugation with therapeutic payloads. In one study, the 3′-end of the aptamer was functionalized with a propargyl adenosine, allowing for covalent linkage to a small peptide via copper-catalyzed click chemistry. This conjugation was achieved using N,N,N′,N′,N″-pentamethyldiethylenetriamine (PMDETA) as a stabilizing agent, ensuring the preservation of the RNA’s structural integrity by avoiding harsh solvents like DMSO [94].

The therapeutic potential of Gint4.T was demonstrated in a study where it was conjugated to a mimetic peptide (MP) designed to modulate the L-type calcium channel (LTCC) in cardiomyocytes. The LTCC, which includes the pore-forming Cavvα1.2 subunit and the regulatory Cavvβ2 subunit, is critical for cardiac contractility, and its dysfunction is linked to various cardiovascular pathologies [95]. The Gint4.T-MP conjugate was shown to effectively restore Cavvα1.2 protein levels and LTCC-dependent calcium currents in HL-1 cardiomyocytes under stress conditions. Importantly, the aptamer-mediated delivery of MP achieved results comparable to those of a cell-penetrating peptide (R7W-MP) but with the added advantage of cell specificity, minimizing off-target effects [13].

Despite these promising findings, challenges remain in the clinical translation of Gint4.T-based therapies. One limitation is the expression of PDGFRβ in non-cardiac cell types, such as fibroblasts, which could lead to unintended effects. To address this, future research could focus on identifying aptamers with higher selectivity for cardiomyocyte-specific markers. Additionally, further preclinical studies are needed to evaluate the long-term safety and efficacy of aptamer-peptide conjugates in animal models of heart disease.

The development of Gint4.T as a delivery vehicle represents a significant advancement in cardiac therapeutics. Its success in preclinical models highlights the potential of aptamer-based strategies for treating conditions like heart failure and arrhythmias. Moving forward, efforts should concentrate on optimizing cardiac targeting, scaling up production under good manufacturing practices (GMP), and advancing toward early-phase clinical trials. The integration of aptamer technology with peptide therapeutics opens new avenues for precision medicine in cardiology, offering hope for more effective and safer treatments for cardiovascular diseases.

Key references supporting these findings include studies by Camorani et al. [93], which characterized the PDGFRβ-binding properties of Gint4.T, and Romanelli et al. [13], which demonstrated its application in cardiac peptide delivery. Further insights into the role of PDGFRβ in cardiac physiology were provided by Chintalgattu et al. [96], underscoring its relevance as a therapeutic target. Together, these studies lay the groundwork for future innovations in aptamer-based cardiovascular therapies.

Receptor-Mediated Internalization: An RNA aptamer (Gint4.T), conjugated to a mimetic peptide (MP), binds PDGFRβ, enabling endocytosis and intracellular cargo delivery.

Intracellular Target Engagement: The cell-penetrating RNA aptamer (RNA-Apt30) binds unphosphorylated phospholamban (PLN) at the sarcoplasmic reticulum (SR). This disrupts PLN’s inhibition of SERCA2a, enhancing Ca^2+^ reuptake into the SR to restore calcium homeostasis.

### 5.2. RNA-Apt30 (Against Phospholamban, PLN)

- Target: Phospholamban, a regulatory protein for the calcium pump (SERCA2a) in cardiomyocytes.
- Application: RNA-Apt30 binds to unphosphorylated PLN, relieving its inhibition of SERCA2a. This improves calcium reuptake and enhances cardiac contractility, showing promise for heart failure therapy (Figure 4).

The sarco(endo)plasmic reticulum Ca^2+^-ATPase 2a (SERCA2a) and its inhibitory regulator phospholamban (PLN) play a central role in calcium handling in cardiomyocytes, with their dysfunction being a hallmark of heart failure (HF) [97]. In HF, reduced SERCA2a activity due to excessive PLN inhibition leads to impaired calcium reuptake into the sarcoplasmic reticulum, resulting in diminished contractility and delayed relaxation [98]. Traditional approaches to modulate this system, such as β-adrenergic agonists, are limited by side effects and desensitization, while gene therapy strategies like SERCA2a overexpression face delivery challenges [99]. In this context, RNA aptamers have emerged as promising tools due to their high specificity, modularity, and lack of immunogenicity.

One such aptamer, RNA-Apt30, was developed through systematic evolution of ligands by exponential enrichment (SELEX) against the cytoplasmic domain of human PLN [14]. Derived from a 40-nucleotide library with a phosphorothioate-modified backbone for enhanced stability, RNA-Apt30 was truncated to a 30-nucleotide oligomer without losing functionality. This aptamer exhibits high affinity for unphosphorylated PLN (Kd = 11 nM) but does not bind phosphorylated PLN or phosphomimetic mutants like S16E-PLN, demonstrating its specificity for the active inhibitory form of PLN. Mechanistically, RNA-Apt30 binds to PLN’s cytoplasmic domain, relieving its suppression of SERCA2a and thereby enhancing calcium reuptake into the sarcoplasmic reticulum. In isolated cardiac SR vesicles, RNA-Apt30 increased SERCA2a activity with an EC50 of 18 nM, comparable to the effects of PLN phosphorylation by protein kinase A. To enable intracellular delivery, RNA-Apt30 was conjugated to a cell-penetrating peptide (CPP), allowing efficient uptake into adult rat cardiomyocytes. Once internalized, the aptamer-enhanced Ca^2+^ transient amplitude and improved contractility, even in the presence of the β-blocker propranolol, suggesting its potential utility in HF patients with compromised adrenergic responsiveness. This is particularly significant given the limitations of current HF therapies, which often fail to address underlying calcium handling defects. Unlike gene therapy approaches, such as those explored in the CUPID (Calcium Up-Regulation by Percutaneous Administration of Gene Therapy in Cardiac Disease) trial with SERCA2a adenoviral delivery [100], RNA-Apt30 acts post-translationally, avoiding risks associated with viral vectors and genomic integration.

While RNA-Apt30 shows great promise, several challenges remain. The phosphorothioate modifications, while improving nuclease resistance, may still require optimization for long-term stability in vivo. Additionally, the reliance on CPP-mediated delivery raises questions about cardiomyocyte specificity and potential off-target effects in other cell types. Future studies should explore tissue-targeted delivery systems, such as lipid nanoparticles or cardiac-specific aptamer conjugates, to enhance therapeutic precision [75]. Preclinical studies in large animal models of HF are also needed to validate efficacy and safety before clinical translation.

In comparison to other aptamers in cardiology, such as the PDGFRβ-targeting Gint4.T for cardiac peptide delivery [13], RNA-Apt30 represents a distinct approach by directly modulating a key regulatory complex in calcium cycling. Its development aligns with growing interest in targeting SERCA2a-PLN interactions, as seen with small molecules like istaroxime, which combines SERCA2a activation and Na^+^/K^+^-ATPase inhibition [101]. However, aptamers offer unique advantages, including the ability to fine-tune specificity and minimize off-target effects through rational design.

## 6. Advanced Technological Platforms Based on Aptamers and Nanomaterials

### 6.1. Theranostics and Drug Delivery

#### Liposomes and Polymeric Nanoparticles (e.g., PEG-PLGA)

**Figure 5:**
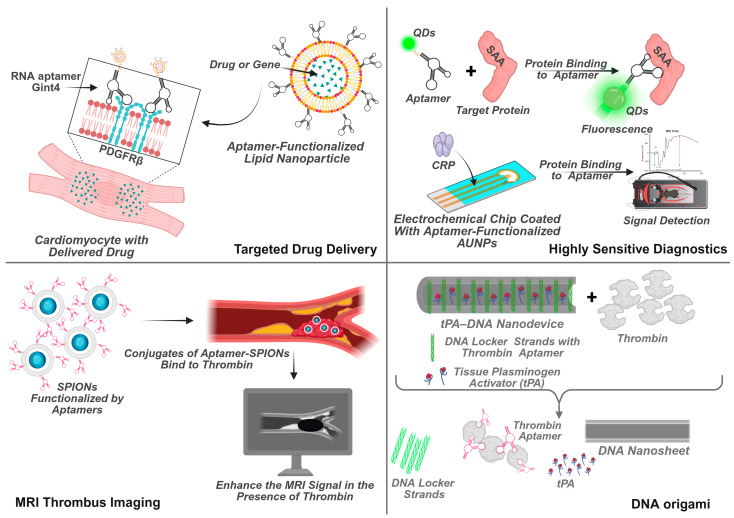
Aptamer-Functionalized Nanoplatforms for Cardiology. Schematic overview of four advanced nanomaterial classes engineered with aptamers for targeted therapy and diagnosis. Created in BioRender. Berezovski, M. (2026) https://BioRender.com/zykb2xk (accessed on 18 February 2026).

Aptamers immobilized on the surface of nanoparticles enable targeted delivery of therapeutic nucleic acids (mRNA, siRNA) and drugs directly to heart cells, minimizing side effects (Figure 5).

The figure illustrates four key strategies:

Targeted Drug Delivery: A lipid/polymer nanoparticle is decorated with aptamers (e.g., Gint4.T) for specific receptor binding (e.g., PDGFRβ) on cardiomyocytes, enabling precise delivery of encapsulated drugs or nucleic acids.

Highly Sensitive Diagnostics: Quantum dots (QDs) and gold nanoparticles (AuNPs) are functionalized with biomarker-specific aptamers (e.g., for CRP). Target binding induces a measurable fluorescent (QDs) or colorimetric (AuNPs) signal change.

MRI Thrombus Imaging: Superparamagnetic iron oxide nanoparticles (SPIONs) are coated with thrombus-targeting aptamers. Their accumulation at a thrombus site within a vessel creates a local signal hypointensity, enabling visualization on a schematic MRI scan.

Logic-Gated Therapeutic Release (DNA Origami): A programmable DNA origami nanostructure acts as a scaffold. It presents multiple thrombin-binding aptamers that function as “locks” on a DNA nanosheet loaded with tissue plasminogen activator (tPA). At a thrombus site, high local thrombin concentrations trigger aptamer binding, unlocking the structure to release tPA precisely, thereby minimizing systemic bleeding risk.

Liposomes are known as the most-studied and widely used non-viral vectors for therapeutic nucleic acid delivery [102]. Liposomes are spherical vesicles composed of lipid bilayers encapsulating hydrophilic and hydrophobic drugs [103]. Their biocompatibility, ability to mimic biological membranes, and versatility in drug encapsulation make them an ideal platform for delivering cardiovascular therapeutics [104]. Lipids have a structure similar to phospholipid bilayers and are generally composed of three parts: a hydrophilic domain, a hydrophobic domain, and a bridging domain (linker) [105]. Hydrophilic domains usually have one or more positive charges and can interact with negatively charged nucleic acids to form liposome/nucleic acid complexes that protect the nucleic acid from nuclease degradation. Hydrophobic domains are usually composed of steroidal compounds or alkyl chains and have a great influence on the efficiency of nucleic acid delivery. The bridging domains usually take glycerol as the skeleton and connect the hydrophilic and hydrophobic domains with amide, ester, or ether bonds. In general, the ester bonds are able to be biodegraded during systemic circulation. Therefore, the ester bond-linked liposomes have higher release efficiency and less cytotoxicity [102]. Generally, the stability, membrane fusion, and transfection efficiency of lipids alone after interaction with DNA are very poor, so neutral auxiliary lipids are often needed [106]. Cationic liposomes formed by mixing cationic lipids and neutral auxiliary lipids at a certain molar ratio can greatly improve transfection efficiency.

In order to deliver nucleic acid to specific cells and reduce the toxicity and side effects on normal cells, specific ligands can be modified on the surface of liposome nanoparticles (LPNs) to bind with specific receptors on the surface of cells and enter cells through receptor-mediated effects. The ligands used to modify liposomes include folic acid, galactose, choleric acid, peptide, and nucleic acid aptamers [102]. In the beginning, the LNP platform focused on siRNA delivery. They have been used for the delivery of the first FDA-approved siRNA therapeutic, Patisiran (Onpattro, Alnylam, Inc., Cambridge, MA, USA) in 2018 for treatment of hereditary amyloidogenic transthyretin (ATTR) amyloidosis [107]. Later, the LNP delivery system was shifted toward the delivery of mRNA and opened the way for the clinical use of LNP-based COVID-19 mRNA vaccines, including Tozinameran (Comirnaty) for Pfizer–BioNTech and Elasomeran (Spikevax) for Moderna [108]. Great prospects are associated with LPN-mediated mRNA delivery in cardiovascular diseases [109]. mRNA emerges as a promising therapeutic agent due to its versatility in encoding therapeutic proteins and targeting “undruggable” conditions. Liposome-delivered nucleic acid-based therapies act through therapeutic protein expression, regulation, gene editing, or silencing of pathogenic genes for the treatment of myocardial ischemia, heart failure, and hypercholesterolemia.

Aptamers are emerging as versatile carriers for targeted delivery of therapeutic payloads to cardiac cells. Gint4.T enables receptor-mediated delivery of peptides to cardiomyocytes via PDGFRβ, while RNA-Apt30 directly modulates intracellular phospholamban to enhance calcium handling. These proof-of-concept studies demonstrate the potential for precision medicine in heart failure. However, challenges remain in achieving cardiomyocyte-specific targeting, ensuring long-term in vivo stability, and advancing these candidates toward clinical evaluation in large animal models.

### 6.2. Diagnostic Platforms

- Quantum Dots (QDs), Carbon Nanodots (C-Dots), Gold Nanoparticles (AuNPs): Used in combination with aptamers to create highly sensitive fluorescent, colorimetric, and electrochemical sensors for detecting troponin, CRP, thrombin, and other biomarkers. (Figure 5)
- Graphene and Graphdiyne (GDY): Enhance the sensitivity of electrochemical aptasensors due to their unique conductive properties.
- Magnetic Nanoparticles: Aptamer-functionalized particles serve as contrast agents for MRI-based thrombus imaging. (Figure 5)
- Polymeric Nanoparticles: PEG-PLGA nanoparticles for targeted antiplatelet drug delivery.

Poly(lactic-co-glycolic acid) (PLGA) is a widely used biodegradable and biocompatible copolymer in drug delivery systems [110]. PLGA can be synthesized through direct condensation of lactic acid and glycolic acid or by ring-opening polymerization (ROP) of lactide and glycolide cyclic dimers. PLGA can be modified with functional polymeric blocks such as polyethylene glycol (PEG), forming PEG-PLGA nanoparticles [111]. PEGylation can reduce immunogenicity and protect drugs from degradation and elimination [112]. A new nano delivery system based on PEG-PLGA nanoparticles coated with nonpeptide platelet glycoprotein IIb/IIIa receptor antagonist Tirofiban [113] for preventing early thrombosis in vein graft was presented by Gao et al. [114].

#### 6.2.1. Quantum Dots (QDs) and Upconverting Nanoparticles (UCNPs)

Fluorescent biosensors for C-reactive protein (CRP) and other biomarker detection.

The excellent optical properties of quantum dots (QDs) make them an ideal fluorescent probe for multiplexed detection. (Figure 5) Gao et al. [15] constructed 10.5 nm CdSe/ZnS QDs incorporated DMSNs-QDs for C-reactive protein (CRP) detection, which achieved a limit of detection of 5 pg mL^−1^. Some common metal ions are known to affect the stability and fluorescence properties of QDs, but scarcely any systematic research has been done about their impacts on QD-based bio-detection. By evaluating the effect of Ca^2+^ metal ions on the properties of aqueous QDs, a new metal ion-QD fluorescence signal amplification sensor (i.e., Ca^2+^-QD-fluorescence-linked immunosorbent assay, Ca^2+^-QD-FLISA) was developed by Lv et al. in 2023 [115] for the detection of inflammatory biomarkers with high sensitivity. The significant improvement in detection sensitivity was achieved due to the crosslinking of aqueous QDs by Ca^2+^ ions to enhance fluorescence and, at the same time, promote antigen–antibody binding efficiency. A dual protection scheme of polymer and silica was proposed by Lv et al. in 2024 [116] to prepare high-quality three-color QD nanobeads (QBs) using three QDs with different ligands. The authors tailored three-color QBs as fluorescent probes based on fluorescence-linked immunosorbent assays (tQBs-FLISA) to detect multiple inflammatory biomarkers simultaneously, including C-reactive protein (CRP), serum amyloid A (SAA), and procalcitonin (PCT). The proposed tQBs-FLISA showed good sensitivity and accuracy without interference from common serum factors. Incorporating QDs into dendritic mesoporous silica nanoparticles (DMSNs) for signal amplification of label materials represented an efficient strategy to improve the performance of lateral flow immunoassays (LFIAs).

#### 6.2.2. Carbon Nanodots (C-Dots)

Carbon nanodots (C-Dots) have attracted growing interest in recent years due to their low cost, ready scalability, excellent chemical and colloidal stability, biocompatibility, and resilience of photoluminescence, serving as environmentally friendly replacements to traditional heavy-metal-based QDs [117]. They are quasispherical nanoparticles with ultrafine dimensions and tunable surface functionalities, for which a sheer variety of simple, fast, and cheap synthesis routes are available [118]. C-Dots have broad excitation spectra, fascinating photoluminescence emission in both solution and solid state, and high stability against photo bleaching and blinking [119]. Due to their low-cytotoxicity, C-Dots have potential applications in biochemical and cell biological fields. An assay with aptamer-functionalized C-Dots as a sensory platform for thrombin detection was presented by Xu et al. [117]. The presence of thrombin can induce the aptamer-modified fluorescent C-Dots to form a sandwich structure with aptamer-functionalized silica nanoparticles (SNPs) through specific protein/aptamer interaction. Thrombin detection was realized by monitoring the fluorescence of TBA29–C-Dots complexes captured by the TBA15–SNPs hybrids due to the formation of aptamer-assembled sandwich SNPs–thrombin–C-Dots. The assay shows high specificity toward thrombin with a detection limit of 1 nM.

#### 6.2.3. Gold Nanoparticles (AuNPs)

Aptamer-conjugated AuNPs for colorimetric/electrochemical sensors.

Biomaterial-metallic nanoparticle hybrid systems are extensively used in different bioanalytical applications [120]. Gold nanoparticles (AuNPs) present high chemical stability, easy and reproducible preparation and surface modification methods, shape and size controllability, and low-toxicity, properties that have attracted substantial attention for several biological applications, including in vitro detection and diagnostics [121,122]. In addition, gold nanoparticles display high surface-to-volume ratios, which contribute to very high loading capacities and lead to improved sensitivity of the analytical system [123]. Most relevant for bio-sensing applications are the unique electrical and optical properties of AuNPs [124]. Single-stranded DNA oligonucleotides of defined length and sequence were first attached to gold nanocrystals via the 5′ end modified by a thiol group in 1996 [125]. The optical properties of Au NPs were employed to follow colorimetric polynucleotide detection. Introduction of a single-stranded target oligonucleotide into a solution containing the nanoparticle-oligonucleotide conjugates resulted in complementary hybridization with the formation of a polymeric network of nanoparticles with a concomitant color change [126].

Amplified detection of thrombin in solution and on surfaces using an aptamer-functionalized AuNP was demonstrated by Pavlov et al. [127]. The plasmon absorbance decreased during AuNPs aggregation and precipitation from the reaction medium after adding different concentrations of thrombin. An optical sensing of thrombin on glass surfaces was conducted by the covalent attachment of the aptamer to a maleimide-functionalized siloxane monolayer, where thrombin was bound to the aptamer interface. The aptamer-functionalized AuNPs were then associated with the second thrombin binding site, resulting in an increased number of Au NP seeds for enlargement. As the surface density of the aptamer-functionalized Au NP is higher, the catalytic deposition of gold on the NPs was enhanced, and this was reflected by the higher glass surface absorbance spectra.

The electrochemical modification of low-cost titanium (Ti) metal substrate with AuNPs was used for the aptamer-based detection of cardiac biomarker troponin I (cTnI) [124]. AuNPs were deposited onto Ti sheets by the potential-step deposition method with high density and homogeneity, and good crystallinity. It was then applied as a transducer to immobilize a thiol-functionalized DNA aptamer via the self-assembled monolayer mechanism for the specific binding of cTnI. The electrochemical aptasensor could detect cTnI in the diluted human serum samples in a linear range of 1–1100 pM with a detection limit of ca. 0.18 pM.

Several reviewed papers have been devoted to methods of detection and quantification of C-reactive protein (CRP), a very important biomarker of infection and inflammation [128]. Aptamer-based CRP-detection assays were used for a capacitive label-free biosensor based on charge distribution under the applied frequency by non-Faradaic impedance spectroscopy (NFIS) [129]. An aptamer-antibody sandwich assay detection method of nanoparticle-enhanced surface plasmon resonance was shown with the detection of CRP in diluted human serum in concentrations ranging from 10 pM to 100 nM [55]. Electrochemical assays can use distinct procedures for the detection of CRP, namely amperometric, potentiometric and electrochemical impedance spectroscopy (EIS) [130]. An RNA aptamer-based electrochemical CRP aptasensor composed of amorphous silica microspheres assembled with AuNPs via Au-N bond was demonstrated by Wang et al. [131]. An anti-CRP aptamer was added to AuNPs, and cyclic voltammetry (CV) and EIS measurements were used to confirm the sensitivity of the aptasensor.

#### 6.2.4. Graphene and Derivatives

Graphene oxide (GO) and hydrogenated graphdiyne (HsGDY) for enhanced biosensor sensitivity.

Graphene has shown great promise in the development of biosensing devices and is of increasing interest for the development of ultra-sensitive biosensors due to its single-atom layer thickness, extremely high carrier mobility, unique electrical conductivity, and inherently low electrical noise [132]. Graphene oxide (GO) is an oxidized form of graphene and has an atomically thin sheet-like structure, which contains nanometer-sized graphene-like *sp*^2^ domains [133]. GO is an excellent acceptor for fluorescence resonance energy transfer (FRET) in the entire visible wavelength region, making GO a promising material for FRET-based biosensors. Its strong molecular adsorption via a π–π interaction provides a high affinity to aptamers. A FRET-based approach was proposed for multiple protein detection and was proven as a sensitive GO surface on-chip aptasensor for thrombin and prostate-specific antigen (PSA) detection by using thrombin and PSA-binding dye-conjugated DNA aptamers [134]. The aptamer terminus opposite the dye-labeled end was firmly fixed to the GO surface by a pyrene linker, and the dye was located close to the GO surface, quenching its fluorescence. If a target was presented in the sample, the dye-labeled aptamer formed a complex with the target and left the GO surface, recovering the fluorescence.

Graphdiyne (GDY), as a novel two-dimensional carbon material, showcases immense potential in the field of smart materials due to its intrinsic properties and microstructure. Unlike conventional smart materials, GDY exhibits stimulus-responsive behaviors without the need for external chemical modifications, dopants, or composite materials. Its unique *sp/sp*^2^ hybridized carbon framework, porous structure, and abundance of highly reactive acetylenic linkages enable this material to directly interact with environmental stimuli and exhibit superior performance across a variety of applications, including biomedical applications [135]. Gu et al. [136] utilized the unique *sp/sp*^2^ hybridized carbon in GDY to develop a hemin/GDY-based biosensing platform for the sensitive detection of glutathione. Niu et al. [137] introduce a one-step in situ chemical etching method to create Cu quantum dots (QDs) coated with GDY nanosheets. The biosensor, constructed by sequentially depositing Cu@GDY, glutaraldehyde, and acetylcholinesterase (AChE), effectively detects organophosphates (OPs) by monitoring AChE inhibition and can sensitively determine glucose without enzymes. Wang et al. [138] developed a photoelectrochemical biosensing platform using GDY and cadmium sulfide quantum dot complexes (GDY-CdSQDs) for the detection of the cancer marker microRNA-21, taking advantage of the excellent photoelectrochemical properties of GDY. In the presence of microRNA-21, the DNA probe molecule selectively hybridizes with the target and binds to DNA probe 2 (P2) on the GDYO nanosheet (P2-GDYO), leading to significant changes in photocurrent and enabling quantitative analysis of microRNA-21. Yao et al. [139] used a novel photoelectrochemical (PEC) sensing method developed using an AuNPs/GDY composite electrode and a WSe2 nanoflower to sensitively detect α-synuclein (α-Syn) with a limit of detection of 3.3 aM. Wang et al. developed a heteronanostructure combining hydrogen-substituted graphdiyne (HsGDY) with nanodiamonds (NDs) for electrochemical aptasensing of acute myocardial infarction markers [140]. The HsGDY@NDs-based aptasensor achieved remarkably low detection limits of 6.29 fg mL^−1^ for cardiac cTnI and 9.04 fg mL^−1^ for myoglobin, with excellent selectivity and performance in human serum samples. This work demonstrates the significant potential of GDY-based nanomaterials for sensitive cardiovascular diagnostics.

#### 6.2.5. Magnetic Nanoparticles

Aptamer-functionalized nanoparticles for MRI-based thrombus imaging.

Due to strong and specific binding capacities, aptamers can serve as escort molecules and can be applicable in targeted imaging diagnostics (Figure 5) [141]. Magnetic Resonance Imaging (MRI) allows the non-invasive visualization of internal structure and soft tissue morphology by the use of a powerful magnet and radiofrequency energy [142]. Targeted imaging research for better delineation of anatomical structures and differentiation between normal and pathological tissues is currently dominated by ligand-modified contrast media for applications in MRI. Aptamers offer several advantages over other commonly used targeting ligands due to their small size, nonimmunogenic nature, tight and specific target binding, and ease of synthesis and handling [143]. One category of aptamer-based MRI contrast agents or probes is based on the relaxation time change caused by conformational alteration of aptamers during their interactions with their targets. A “turn-on” MRI agent using superparamagnetic iron oxide nanoparticles (SPIONs) functionalized by aptamers was introduced by Yigit et al. in 2007 [144]. The conjugation of aptamers with their protein targets was efficient to dephase the spins of neighboring water protons, leading to an alteration in the spin-spin relaxation time (T2). An MRI contrast agent based on aptamer-conjugated SPIOs for the detection of thrombin was presented by Yigit et al. in 2008 [145]. The aptamer-functionalized nanoparticles were able to form an assembly and enhance the MR signal in the presence of thrombin. A detectable change in MRI signal is observed with 25 nM thrombin in human serum.

### 6.3. Smart Photonic Hydrogel for Serum Thrombin Detection

Smart photonic hydrogels are a promising platform for developing novel chemical and biological sensors due to their facile optical signal readout and highly sensitive responsivity toward target analytes [146]. Two-dimensional photonic hydrogels are fabricated by embedding a two-dimensional photonic crystal (2DPC) into a polymer hydrogel network [147]. Two partially base complementary aptamer-functionalized 2DPC hydrogels as aptasensors for the detection of thrombin in human serum were developed by Snen et al. [148]. Amino-terminated DNA aptamers were linked into a carboxyl-rich hydrogel network containing a polystyrene 2DPC array by amide bonds. Upon exposure to thrombin solution, the photonic hydrogel aptasensors swelled, and the particle spacing of the 2DPC embedded in the hydrogel networks increased. The binding between thrombin and one of the aptamers opened the complementary bases of the DNA strands linked to the polymer chains, releasing the other aptamer and leading to a decrease in the cross-linking density of the hydrogel. The particle spacing changes were acquired by simply measuring the diameters of the Debye ring diffracted by the 2DPCs without the requirement of sophisticated instruments. The particle spacing increase in the optimized aptasensor was linear over the thrombin concentration range of 1–500 nM, and the limit of detection was 0.64 nM.

### 6.4. Hybrid Systems

DNA Origami: Nanoassemblies for thrombin binding.

DNA origamis are formed by the assembly of multiple short ssDNA staples onto a long ssDNA, thereby creating 2D or 3D structures. Based on Watson–Crick hydrogen bonding, these structures can range from several nanometers to sub-micrometers range. Compared to duplex DNA structures, the DNA origamis exhibit different mechanical properties in terms of robustness, elasticity, and degradability [149]. Through the DNA origami technique, DNA undergoes a transformation from a self-assembled one-dimensional (1D) double helix to multidimensional structures, unlocking potential functional applications. The resulting nanostructures consist of hundreds of different staples and a long scaffold, offering excellent addressability and programmability [150]. Several studies have used thrombin binding aptamer (TBA) to embed into DNA origami structures for testing and prospective use in medical applications. Tintoré et al. developed a biosensor to visualize human O6-alkylguanine-DNA alkyltransferase (hAGT) activity on an origami-based platform [151]. Their work applied single-molecule characterization of DNA origami for DNA repair assays by combining α-thrombin with TBA to determine the repair effect of hAGT, a DNA-binding protein responsible for the repair of the O6-methylguanine, contributing to the resistance to chemotherapeutic agents. They designed a DNA origami in which some of the staple strands were modified by the insertion of TBA1 and TBA2 in the middle, protruding from the DNA origami surface [152]. The staple strands were arranged asymmetrically along the length of the origami in a way that allowed the differentiation between methylated/non-methylated, to enable the observation and quantification of α-thrombin interaction with the aptamers. Atomic force microscopy was used for visual binding detection. The complex between the dual-aptamer system and α-thrombin is only formed with the non-methylated TBA, and confirms the ability of the design to discern between the methylated and non-methylated state. Kosinski et al. used thrombin-binding aptamers to link thrombin to various DNA nanostructures for the study of DNA/substrate electrostatic interactions [153]. They compared freely diffusing aptamers in solution with TBAs, anchored to a DNA origami scaffold. The catalytic reaction rate was affected by DNA/substrate electrostatic interactions, proportionally to the degree of DNA/enzyme tethering. For substrates of opposite net charge, this led to an inversion of the catalytic response of the DNA-scaffolded thrombin when compared to its freely diffusing counterpart. The DNA nanostructures interfered with charge-dependent mechanisms of enzyme-substrate recognition, altering the electrostatic environment near the encaged enzyme. Zhao et al. described DNA origami-based assemblies that enable the inhibition of thrombin activity and thrombus formation [16]. Two different thrombin-binding aptamers decorated DNA-origami initiated protein recognition and inhibition, exhibiting enhanced anticoagulation in human plasma, fresh whole blood and a murine model. In a dialyzer-containing extracorporeal circuit that mimicked clinical hemodialysis, the origami-based aptamer nanoarray effectively prevented thrombosis formation. Oligonucleotides containing sequences complementary to the thrombin-binding aptamers efficiently neutralized the anticoagulant effects. Khoshouei et al. incorporated thrombin binding aptamer (TBA) into the multilayer DNA origami scaffold and resolved its overall shape using cryogenic electron microscopy (cryo-EM) single-particle analysis [154]. Krissanaprasit et al. presented a safe and effective coagulation control system with a fast-acting, specific reversal functional RNA origami-based agents [155]. It consisted of an RNA origami-based direct thrombin inhibitor (HEX01), containing multiple thrombin-binding aptamers, and a new single-molecule DNA antidote (HEX02), reversing the anticoagulation activity of HEX01 in human plasma within 30 s in vitro. HEX01 molecule contains two copies of each exosite-1- binding aptamer R9D-14T [156] and exosite-2-binding RNA aptamer Toggle-25t [157] located at each end of the two double helices and can simultaneously bind two thrombin molecules. The DNA antidotes with aptamer unfolding abilities were constructed as complementary strands.

Yin et al. [158] described an intelligent DNA nanodevice for precision thrombolysis. They presented a method for precise delivery and accurate dosing of tissue plasminogen activator (tPA) using a DNA origami to integrate DNA nanosheets with predesigned tPA binding sites and thrombin-responsive DNA fasteners (Figure 5). The fastener is an interlocking DNA triplex structure that acts as a thrombin recognizer, threshold controller and opening switch. When loaded with tPA and intravenously administered in vivo, these DNA nanodevices rapidly targeted the site of thrombosis, tracked the circulating microemboli and exposed the active tPA only when the concentration of thrombin exceeded a threshold. The authors demonstrated the method with improved therapeutic efficacy in ischaemic stroke and pulmonary embolism models, supporting the potential of these nanodevices to provide accurate tPA dosing for the treatment of different thromboses.

### 6.5. Hybrid Nanomaterials

Nanodiamond-graphdiyne composites for cTnI/Mb detection.

A new type of crystalline porous material, covalent organic frameworks (COFs) are constructed with organic building blocks containing light elements (e.g., C, N, O, H, and B) and linked by strong covalent bonds [159]. COFs possess large specific surface areas, porous and predictable crystalline structures, tunable functionality, low density, and mechanical robustness [160]. Among many kinds of COFs, cross-linked carbon-rich graphdiyne frameworks synthesized through multiple alkyne-alkyne dimerization of hexaethynylbenzene possess a highly extended π-conjugated system [160]. HsGDY shows satisfactory conductivity and ion diffusion and has been applied in diverse fields [161]. NDs are carbon-based nanomaterials that show good biocompatibility and consist of nanosized tetrahedral networks. NDs have been applied to biosensing [162] and biomedical fields [163]. A nanohybrid of HsGDY and NDs was applied as the scaffold for anchoring aptamer strands to fabricate electrochemical aptasensors for detecting the acute myocardial infarction biomarkers myoglobin and cardiac troponin I [140]. These aptasensors exhibited high selectivity, good stability, reproducibility, and acceptable applicability in real human serum, broadening the application of porous organic frameworks in the sensing field and providing a prospective approach for the early detection of cardiac disease biomarkers.

Integration of aptamers with nanomaterials—quantum dots, gold nanoparticles, graphene, and DNA origami—has enabled sophisticated theranostic platforms with unprecedented sensitivity and control. Smart systems like thrombin-responsive DNA origami for precision thrombolysis exemplify the shift toward programmable therapeutics. However, clinical translation is hindered by scalability challenges, potential nanomaterial toxicity, and limited in vivo validation. Future efforts should prioritize safety profiling, GMP-compliant manufacturing, and rigorous testing in clinically relevant disease models.

## 7. Conclusions

Aptamers have emerged as a highly versatile molecular tool with significant potential to transform cardiology. Their unique properties—high specificity, low immunogenicity, ease of chemical modification, and reversible action—position them as compelling alternatives to traditional antibodies and small-molecule drugs. In diagnostics, aptamer-based biosensors have demonstrated exceptional sensitivity for detecting key cardiac biomarkers such as troponins, CRP, and natriuretic peptides, enabling early disease detection and point-of-care applications. Therapeutically, aptamers targeting coagulation factors (vWF, thrombin, FIXa) offer precise, controllable anticoagulation with the unique advantage of rapid reversal by specific antidotes. Furthermore, aptamers like Gint4.T and RNA-Apt30 have opened new frontiers in targeted drug delivery and direct modulation of cardiomyocyte function, addressing intracellular targets previously considered “undruggable.” The integration of aptamers with advanced nanomaterials—quantum dots, graphene, DNA origami—has further expanded their utility, creating sophisticated theranostic platforms for both imaging and therapy.

Despite these advances, challenges remain, including the need for enhanced in vivo stability, large-scale GMP production, and strategies to mitigate PEG-related immunogenicity observed in some clinical trials. Nevertheless, the growing number of clinical-stage candidates and the rapid pace of innovation in aptamer chemistry and nanotechnology underscore the immense promise of this field.

## 8. Future Prospects

Looking ahead, several key directions will likely shape the next generation of aptamer-based applications in cardiology:

Discovery of novel aptamers: The application of advanced selection techniques, such as cell-SELEX and in vivo SELEX, will facilitate the discovery of aptamers against complex membrane receptors and previously intractable targets. Furthermore, the integration of artificial intelligence and machine learning for in silico aptamer design and optimization promises to accelerate the development pipeline and improve binding affinity and specificity.

Enhanced in vivo stability and delivery: Future efforts will focus on developing next-generation chemically modified aptamers (e.g., Spiegelmers, thiophosphate backbones) with superior resistance to nuclease degradation and prolonged half-lives. For intracellular targets, the development of safer and more efficient delivery vehicles, such as cell-penetrating peptides, lipid nanoparticles, and cardiac-targeted adeno-associated viruses carrying aptamer sequences, will be critical for clinical translation.

Overcoming immunogenicity: A major lesson from the REG1 system is the critical need to address PEG-related immunogenicity. Future therapeutic aptamers will likely move away from PEGylation, exploring alternative conjugation strategies with biocompatible polymers (e.g., zwitterionic polymers, hydroxyethyl starch) or the use of fully modified nucleotides that are less recognizable by the immune system.

Spatially controlled and logic-gated therapies: Inspired by advances in DNA nanotechnology, we anticipate the development of smarter therapeutic platforms. “Logic-gated” DNA origami devices, such as the thrombin-responsive tPA delivery system, represent a paradigm shift. Future iterations could incorporate multiple disease-specific biomarkers to trigger drug release only in precisely defined pathological microenvironments, thereby maximizing efficacy and minimizing off-target effects.

Multiplexed diagnostics and theranostics: The combination of aptamers with microfluidic “lab-on-a-chip” technologies will enable rapid, simultaneous, and cost-effective point-of-care detection of multiple cardiovascular biomarkers from a single drop of blood. Moreover, theranostic nanoparticles integrating an aptamer for targeting, an imaging agent for diagnosis, and a therapeutic payload for treatment hold immense potential for personalized cardiovascular medicine, allowing for real-time monitoring of drug delivery and therapeutic response.

In summary, the field of aptamer-based cardiology is poised for significant expansion. By addressing current challenges and harnessing innovations in chemistry, nanotechnology, and data science, aptamers are well-positioned to bridge the gap between precise diagnosis and targeted therapy, ultimately contributing to more effective and personalized interventions for patients with cardiovascular diseases.

## Figures and Tables

**Table 1:** Aptamers targeting cardiac troponin I (cTnI) and troponin T (cTnT) with key characteristics.

Aptamer Name, Target, Length	Aptamer Sequence, 5′-3′ Direction	Type	Kd, (nM)	Method for Affinity Estimation	Reference
TnIApt11, cTnI, 79 nt	GCCTGTTGTGAGCCTCCTAACTTCAAGGTGT GGTCAGTCTTGGATTGGAGGAGTATGNGCAT GCTTATTCTTGTCTCCC	DNA	10.25	Fluorescence	[6]
TnIApt18, cTnI, 79 nt	GCCTGTTGTGAGCCTCCTAACTACATGTTCTC AGGGTTGAGGCTGGATGGCGATGGTGGCATG CTTATTCTTGTCTCCC	DNA	9	Fluorescence	[6]
TnIApt19, cTnI	No sequence published	DNA	6.3	Fluorescence	[6]
TnIApt23, cTnI	No sequence published	DNA	2.69	Fluorescence	[6]
Apt3, cTnI, 96 nt	CGTACGGTCGACGCTAGCCGGACACCCAAGTC AGACGTGCCCATTATCGCGCGATACGTATTAT TTCTTGCTCGGGGCCACGTGGAGCTCGGATCC	DNA	1.01	ELONA	[7]
Apt6, cTnI, 96 nt	CGTACGGTCGACGCTAGCCCGGAGCGAAG GCG GCCCCGTTTGCGTGCAGCG- TAGTCTGTAGACAACAGTGCT- GTGGGCCACGTGGAGCTCGGATCC	DNA	0.68	ELONA	[7]
cTnI-Apt1, cTnI, 80 nt	GGCAGCAGGAAGACAAGACATGGGTGGCGG GGACG GGGCGATGGGAACTTAGATT GCTAGTGGTTCTGTGGTTGCTCTGT	DNA	61.51	Bioluminescence	[17]
cTnI-Apt2, cTnI, 80 nt	GGCAGCAGGAAGACAAGACAGGCAGTGTCA CGCGCTCAAGGGTGGAGGGGTCGGGGAG GTTGGTTCTGTGGTTGCTCTGT	DNA	42.01	Bioluminescence	[17]
TnAp2t1, cTnI, 40 nt	GGCAGTGTCACGCGCTCAAGGGTGGAGGG GTCGGGGAGGT	DNA	39.06	Bioluminescence	[17]
TnAp2t2, cTnI, 54 nt	AGACAAGACAGGCAGTGTCACGCGCTCAA GGGTGGAGGGGTCGGGGAGGTTGGT	DNA	24.93	Bioluminescence	[17]
Tro4, cTnI 40 nt	CGTGCAGTACGCCAACCTTTCTCATGCGCTG CCCCTCTTA	DNA	270	surface plasmon resonance	[21]
Tro6, cTnI, 40 nt	CGCATGCCAAACGTTGCCTCATAGTTCCCTC CCCGTGTCC	DNA	317	surface plasmon resonance	[21]
Tro 4, cTnT, 40 nt	CGTGCAGTACGCCAACCTTTCTCATGCGCT GCCCCTCTTA	DNA	No data	No data	[22]
Apt-1, cTnT, 71 nt	ATACGGGAGCCAACACCAGGACTAACATTA TAAGAATTGCGAATAATCATTGGAGAGCAG GTGTGACGGAT	DNA	122	surface plasmon resonance	[23]
Apt-2, cTnT, 71 nt	ATCCGTCACACCTGCTCTCCAATGATTATTC GCAATTCTTATAATGTTAGTCCTGGTGTTGG CTCCCGTAT	DNA	190	surface plasmon resonance	[23]

Note: Kd: Dissociation constant (lower values indicate higher affinity); ELONA: Enzyme-Linked Oligonucleotide Assay; DNA: Deoxyribonucleic Acid; Apt: Aptamer; TnI: Troponin I; nt: nucleotides.

**Table 2:** Overview of Clinical Trials for Antithrombotic Aptamers.

Aptamer Name, Sequence	Target	ClinicalTrials.gov Identifier (NCT Number) ^1^	Phase, Status	Trial Participants	Results
BT200, sequence not published	Von Willebrand Factor (vWF) + IbPlatelet Glycoprotein Ib (GPIb)	NCT04103034	Phase 1, completed 2020	112 healthy volunteers	Safety/Tolerability: confirmed. Effect: Selective, dose-dependent vWF inhibition.
		NCT04677803	Phase 2a, completed 2021	19 patients with hemophilia A, 8 with mild, 2 with moderate, 9 with severe	Safety/Tolerability: Confirmed. Multi-dose PK/PD: Characterized in target patient population.
BC 007, sequence not published	Autoantibodies against β1-adrenergic	NCT04192214	Phase 2, completed 2022	30 patients with dilated cardiomyopathy and autoantibodies	Safety/Tolerability: Confirmed. Efficacy/Mechanism: Provided effective, long-term neutralization of autoantibodies.
RB006 5′-GGGAGGACGAUGCGGACCGAAAAGGUUCCUCCC-3 ‘	Blood Clotting Factor IXa (FIXa)	NCT01872572	Phase 1, completed 2009	36 healthy volunteers	Safety/Tolerability: Confirmed. PK/PD: Characterized.
REG1/RB006 + RB007	RB007 Coagulation Factor IXa (FIXa)	NCT00715455	Phase 2a, completed 2008	26 patients with stable ischemic heart disease	Procedural Efficacy: Demonstrated effective anticoagulation control with an available antidote during PCI.
		NCT00932100	Phase 2, completed 2011	640 patients with acute coronary syndrome	Safety/Tolerability: Confirmed versus standard therapy. Efficacy: Demonstrated versus standard therapy.
		NCT01848106	Phase 3, terminated 2014	3232 patients after percutaneous coronary intervention	Status: Prematurely terminated. Reason: Safety (allergic reactions) and lack of efficacy advantage over bivalirudin.
NU172 5′-CGCCTAGGTTGGGTAGGGTGGCCG-3′	Thrombin (FIIa)	NCT00808964	Phase 2, completed 2011	30 patients who underwent CABG	Efficacy: Effective thrombin suppression confirmed in post-CABG patients. Safety: Safe profile confirmed in post-CABG patients.
ARC19499, sequence not published	TFPI (Tissue Factor pathway inhibitor)	NCT01191372	Phase 1, terminated 2011	17 patients with hemophilia	Results not published.
ARC1779	Von Willebrand Factor (vWF) + IbPlatelet glycoprotein Ib (GPIb)	NCT00432770	Phase 1, completed 2007	42 healthy volunteers	Safety/Tolerability: Evaluated. Pharmacokinetics (PK): Characterized.
		NCT00632242	Phase 2, completed 2008	28 patients with thrombotic-thrombocytopenic purpura	Mechanism: Dose-dependent inhibition of vWF activity. Effect: Corresponding inhibition of platelet activity.
		NCT00507338	Phase 2, terminated 2008,300	300 patients with acute myocardial infarction	Status: Trial terminated prematurely. Data Availability: Results remain unpublished.
		NCT00742612	Phase 2, terminated	36 patients who underwent carotid endarterectomy	Efficacy: Did not meet the primary endpoint. Safety/Tolerability: Associated with increased risk of perioperative bleeding and anemia.

Notes: ^1^ Data sourced from ClinicalTrials.gov. FIXa: Factor IXa; vWF: von Willebrand Factor; TFPI: Tissue Factor Pathway Inhibitor; CABG: coronary artery bypass grafting. The REG1 system consists of the aptamer RB006 (pegnivacogin) and its complementary antidote RB007 (anivamersen).

## Data Availability

No new data were created or analyzed in this study.

## Abbreviations

The following abbreviations are used in this manuscript:

1D	One-dimensional
2D	Two-dimensional
2DPC	Two-dimensional photonic crystal
3D	Three-dimensional
α-Syn	α-Synuclein
AMI	Acute myocardial infarction
ATTR	Amyloidogenic transthyretin
AuNP	Gold nanoparticle
BNP	Brain natriuretic peptide
CABG	Coronary artery by-pass grafting
C-Dot	Carbon nanodot
CK	Creatine kinase
CK-MB	Creatine kinase-MB isoenzyme
COF	Covalent organic framework
CPP	Cell-penetrating peptide
CRP	C-reactive protein
cryo-EM	Cryogenic electron microscopy
cTnI	Cardiac troponin I
cTnT	Cardiac troponin T
CV	Cyclic voltammetry
CVD	Cardiovascular disease
CUPID	Calcium Up-Regulation by Percutaneous Administration of Gene Therapy in Cardiac Disease
DMSO	Dimethyl sulfoxide
DMSN	Dendritic mesoporous silica nanoparticle
DNA	Deoxyribonucleic acid
DPV	Differential pulse voltammetry
EC50	Half-maximal effective concentration
EIS	Electrochemical impedance spectroscopy
ELISA	Enzyme-linked immunosorbent assay
ELONA	Enzyme-linked oligonucleotide assay
FIXa	Factor IXa
FLISA	Fluorescence-linked immunosorbent assay
FRET	Fluorescence resonance energy transfer
GDY	Graphdiyne
GMP	Good manufacturing practice
GO	Graphene oxide
GPIb	Glycoprotein Ib
hAGT	Human O^6^-alkylguanine-DNA alkyltransferase
HF	Heart failure
Hs-CRP	High-sensitivity C-reactive protein
HsGDY	Hydrogen-substituted graphdiyne
Kd	Dissociation constant
LDL-C	Low-density lipoprotein cholesterol
LFIA	Lateral flow immunoassay
LNP	Lipid nanoparticle
LOD	Limit of detection
LPN	Liposome nanoparticle
LTCC	L-type calcium channel
MRI	Magnetic resonance imaging
mRNA	Messenger RNA
MP	Mimetic peptide
NDs	Nanodiamonds
NFIS	Non-Faradaic impedance spectroscopy
NT-proBNP	N-terminal pro-brain natriuretic peptide
OPN	Osteopontin
PCT	Procalcitonin
PD	Pharmacodynamics
PDGFRβ	Platelet-derived growth factor receptor beta
PEC	Photoelectrochemical
PEG	Polyethylene glycol
PEG-PLGA	Polyethylene glycol-poly(lactic-co-glycolic acid)
PK	Pharmacokinetics
PLGA	Poly(lactic-co-glycolic acid)
PLN	Phospholamban
PMDETA	N,N,N′,N″,N″-Pentamethyldiethylenetriamine
QB	Quantum dot nanobead
QD	Quantum dot
RNAi	RNA interference
ROP	Ring-opening polymerization
SAA	Serum amyloid A
SELEX	Systematic Evolution of Ligands by Exponential Enrichment
SERCA2a	Sarco(endo)plasmic reticulum Ca^2+^-ATPase 2a
SERS	Surface-enhanced Raman spectroscopy
siRNA	Small interfering RNA
SNP	Silica nanoparticle
SPION	Superparamagnetic iron oxide nanoparticle
SPR	Surface plasmon resonance
SR	Sarcoplasmic reticulum
sST2	Soluble ST2
SWV	Square wave voltammetry
TBA	Thrombin-binding aptamer (HD1)
TFPI	Tissue factor pathway inhibitor
tPA	Tissue plasminogen activator
TTP	Thrombotic thrombocytopenic purpura
vWF	von Willebrand factor
